# Spreading Effect in Industrial Complex Network Based on Revised Structural Holes Theory

**DOI:** 10.1371/journal.pone.0156270

**Published:** 2016-05-24

**Authors:** Lizhi Xing, Qing Ye, Jun Guan

**Affiliations:** 1 Department of Business Administration, School of Economics and Management, Beijing University of Technology, Beijing, China; 2 Department of Finance, School of Economics and Management, Beijing University of Technology, Beijing, China; IFIMAR, UNMdP-CONICET, ARGENTINA

## Abstract

This paper analyzed the spreading effect of industrial sectors with complex network model under perspective of econophysics. Input-output analysis, as an important research tool, focuses more on static analysis. However, the fundamental aim of industry analysis is to figure out how interaction between different industries makes impacts on economic development, which turns out to be a dynamic process. Thus, industrial complex network based on input-output tables from WIOD is proposed to be a bridge connecting accurate static quantitative analysis and comparable dynamic one. With application of revised structural holes theory, flow betweenness and random walk centrality were respectively chosen to evaluate industrial sectors’ long-term and short-term spreading effect process in this paper. It shows that industries with higher flow betweenness or random walk centrality would bring about more intensive industrial spreading effect to the industrial chains they stands in, because value stream transmission of industrial sectors depends on how many products or services it can get from the other ones, and they are regarded as brokers with bigger information superiority and more intermediate interests.

## Introduction

In last two decades, statistic physics has been wildly adopted in researching on the dynamic and statistic properties embedded in various economic behaviors and data of the global economic infrastructures [1]. The application of methodologies like stochastic dynamics, self-similarity, scale theory etc. has incurred a new interdiscipline subject—econophysics [2]. Under this circumstance, complex network with abundant utilization of statistic physics can be judged as a branch of econophysics in relevant financial and industrial researches.

As results, a large number of theoretical and empirical researches on industrial economics based on complex network have been accomplished in areas of industrial development, structure, association, organization and policy. Chmiel, et al. establishes networks of companies and branches in Poland through bipartite graph theory [3]. Similarly, Inoue, et al investigate a Japan’s patent network focusing on its spatial characteristics based on the same theory [4]. Chang, et al. show that the degree distribution of nodes in a projected sub-network follows the drift power-law in general cooperation as well as in competition networks [5]. Liu, et al. propose to study the development of China’s high-tech park using complex network, and constructed the network of China’s top 100 electronic and IT companies as the basic model [6]. Hou, et al. extend their research fields from monopoly market to macro-reality market to build competitive complex network model targeting logistics enterprises [7]. Li, et al. establish a global nuclear power plant network based on priority queuing network model and simulated its numerical characteristics to reflect its evolution [8]. Yao, et al. model the directed weighted competitive pressure network and made a simulation analysis on the rivalry spread effect over it [9]. Upper analyze the mechanisms of contagion in banking and financial networks [10]. Hu, et al. calculate economic distance matrices based on annual GDP of nine economic sectors from 1995–2010 in 31 Chinese provinces and autonomous regions, and built spatial economic networks through threshold and minimal spanning tree [11]. All above, scholars create various complex network models to describe inter-organization competition and cooperation analyzing differed economic phenomena. The most common method in existing researches on industrial complex networks is the introduction of unweighted and undirected network models similar to the simple physical ones, with less to be known or much to be neglected on the mechanism of informational, material and capital flows between economic entities manifested in their interdependencies.

From an empirical perspective, a handful of studies have characterized the structure of input-output (later referred to as IO) networks to better understand the topology of inter-industry dependencies and their repercussions on the industrial economics. For instance, Blöchl, et al. adopt STAN database at OECD to establish 37 countries’ IO networks and derive two measures for weighted and directed network, which are, random walk centrality to reveal the most immediately affected nodes by a shock based on Freeman’s closeness centrality, and counting betweenness to identify the most accumulatively affected nodes based on Newman’ random walk betweenness [12]. Kagawa, et al. propose an optimal combinatorial method to find industries with large CO_2_ emissions through industrial relations based on IO table, depicting environmentally important industrial clusters in Japanese automobile supply chain [13]. McNerney, et al. study the structure of inter-industry relationships using networks of capital flows between industries in 20 national economies, and found these networks vary around a typical structure characterized by a Weibull link weight distribution [14]. Martha, et al. investigate how economic shocks propagate and amplify through the IO network connecting industrial sectors in developed economies [15].

With the development of IO database, related researches are not only based on independent national systems but also on multi-regional even global systems, and most of them adopted World Input-Output Database (WIOD) as the data source. For instance, Ando measures the importance of industrial sectors under the impact of American gross output in the global IO model [16]. Cerina, et al. analyze the subgraph structure and dynamics attributions of global network with community detection techniques, pinpointing the key industries and economic entities with PageRank centrality and community coreness [17]. Grazzini and Spelta set up the cost effect index to testify the robustness of global IO network and the interdependency of intermediate inputs in production [18].

The present researches mainly mine the IO data from different aspects as an econophysics context implied in the form of networks, but restricte to static analyzing endogenous variables ignoring the process of fining and refining of variables to maintain equilibrium, let alone providing measurements and advises on optimal control of the evolution tendency of industrial structures.

## Methods

### Impacts of Structural Holes

Structural holes theory is developed by Ronald Stuart Burt and applied in Social Network Analysis [19]. This theory spans the fields of sociology, economics, and computer science. Burt introduced this concept in attempt to explain the origin of differences in social capital, and found positional advantage or disadvantage of individuals result from how they are embedded in neighborhoods. For instance, individuals located on important transmission paths serve as brokers receiving non-redundant information from their contacts. In this way, they are granted with information superiority and possess advantages on top of gaining intermediate interests by connecting the other nodes.

There are two sorts of indices to measure structural holes, which are Burt’s structural holes index and Freeman’s between centrality. The former is mainly applied to evaluate redundancy in ego networks, while the latter is used to calculate important degree of all the nodes. For the whole network, if a node is on many geodesic paths, it actually plays an important intermediate role, usually measured by betweenness. Measured with centrality, if one node enjoys the maximum proximity closeness in a network, implying the minimum summation of geodesic distance, it occupies an important intermediate location.

For this reason, this theory is here introduced to explain the function of industrial sectors in economic circulation, because there exists complex economic independence in the process of industrial division and cooperation.

### Revised Structural Holes Theory

**Fig 1:**
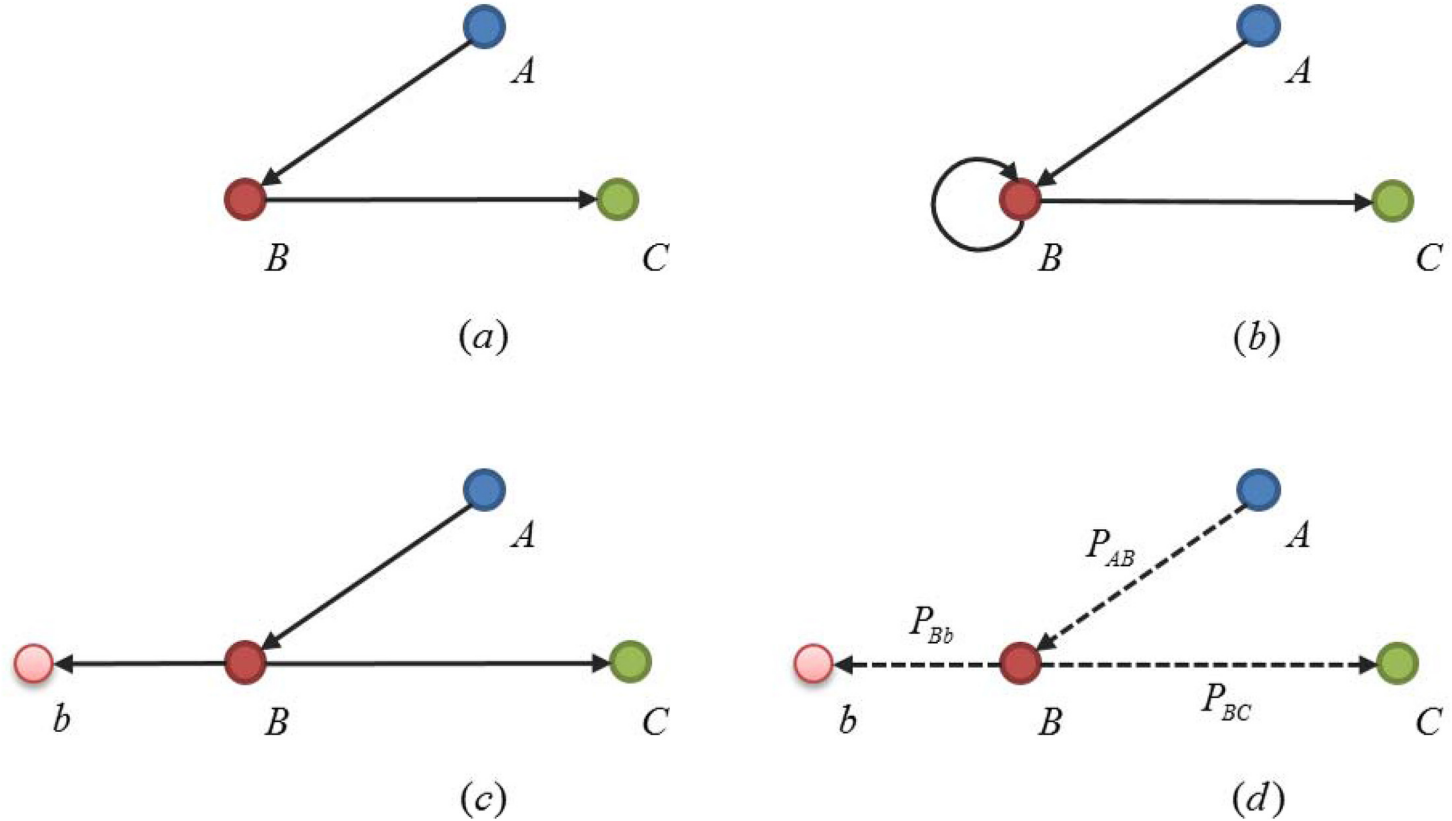
Structural Holes and Its Extension in Complex Social Network Analysis.

In Burt's classic theory, holes come forth in social structures because weak ties exist in relationships between groups on the connections of opportunities, capital, and information. In addition, these holes could also reinforce competitive advantages to the individuals on this relation. As shown in Fig 1(a), *AC* is referred as a structural hole, for there is no (or very weak) direct tie between *A* and *C* in the triangle network. If *A*, *B* and *C* are in the state of resources competition, *B* is to incur information superiority and controlling advantage as an intermediate bridge, for the existence of structural hole between *A* and *C*.

However, there is limitation of its application on complex networks due to its ignorance of weights, directions, dynamics and self-loops, which sabotages its ability on depicting infrastructures of economic systems composed of industrial sectors and the quantitative relationships in between.

If *B* has a self-loop, as shown in Fig 1(b), it is assumed that there is a shadow node *b*, hence self-loop can be replaced by a virtual edge *Bb*, as shown in Fig 1(c). Obviously, shadow nodes will only locate at the tail ends of the network, worth no consideration of their out-degree or out-strength. In view of network flow, they can be regarded as sink nodes for merely receiving from the original ones without any flow outwards.

So the index on between centrality should be modified when applied to study the dynamics of information transmission on networks with the theory of structural holes. The practical value stream transmission in present industrial networks is a Markov process in time-discrete state, taking considerations on not only the direction of value stream, say *B* should have the provision of inflow and outflow of information simultaneously with probabilities of each transmission set respectively, for instance with *P*(*A*, *B*) and *P*(*B*, *C*) referring to the probabilities of existence of information transmission from *A* to *B* and *B* to *C*, but also the worn-out and consumption of output of the industrial sector itself, for instance, the industrial sector should first meet its own needs for products and services with the probability as *P*(*B*, *b*).

### Features of Value Stream in Economic System

Black argues that the fundamental cause of business cycle is the economic impact and its aftermath, which could be defined as the impacts of exogenous variables on the endogenous ones in industrial sectors. Specifically, he categorizes market prices, advances in technology, profit distribution, government policies and final demand as the exogenous variables, and the material and capital flows between different sectors as the endogenous ones [20].

The economic impacts on national economic system constituted by industrial sectors flow along the direction of intermediate inputs, and all of them converge into value stream just like network. The target sectors, in the form of sink nodes on the network, are those whose final demands have been satisfied by the additional input flows at the end of this stochastic process of economic impact. Assuming that for some external reasons, such as government policies, additional productivity in automotive industry becomes an impact or disturbance to the entire economic system, which is to be absorbed by other industries. The abundancy of extra production, on the one hand, is randomly distributed among other sectors, which could be traced from the IO table, and on the other hand, will bring about extra profits in the forms of extra manufacturing funds, labor remuneration and non-direct business taxes for the automotive industry. In this way, the external impacts aroused by automotive industry will be transformed into inputs into other industries. Then, this impact can be shown as domino effects stirring additional economic flows in the economic system, until it finally reaches a new stage of stability with all the impacts and disturbance totally absorbed by other industries.

### Advantage of Input-output Table Date

Beyond all question, IO table as a quantitative technique of economic analysis represents the interdependencies between different branches of a national economy or different regional economies perfectly. Its property of being in the form of checkboard enables it to reflect the movements of products or services within the whole economic system from both production consumption to distributive utilization, which are actually the formation and distribution of values respectively. The dual identities of each industrial sector on the network as the producer and consumer at the same time, demand it not only to produce and distribute providing inputs for the other industrial sectors but also to consume inputs from other sectors to accomplish its own fabrication. This is indeed the inner identity proposed by Karl Marx. The industrial sectors in the IO table could be regarded as nodes while inter-industry value stream contributes to weighted and directed edges in the construction of network models.

Even more important, consideration should be paid on what kinds of impacts value stream bring to each industrial sector in the economic system over different temporal span. On the one hand, IO table depicts the annual inter-industry independence in a nation or region, which reflects the accumulative or long-term effect of value stream. On the other hand, any disturbance as the exogenous variables in the form of supply shocks on one of the economic industrial sector in a stable network will force it make rapid impacts on the neighboring sectors along the existing industrial structure, which reflects the instantaneous or short-term effect of value stream.

## Models

### Underlying Database

In consideration of both availability and authority, IO table is definitely the priority-first data format to establish mathematics model. For instance, it can show flows of final and intermediate goods and services defined according to industry outputs. In addition, it is provided as matrix, which can be directly or with minor modification adopted as complex network’s adjacency matrix, establishing a sort of weighted and directed network.

There are four inter-country IO tables available: World Input-Output Database (WIOD), Structural Analysis Database (STAN), Global Trade Analysis Program (GTAP) and Asian International Input-Output (AIO). WIOD was chosen as underlying database in this paper.

This is because WIOD provides time-series of world IO tables for 40 independent countries, covering the period from 1995 to 2011. These tables have been constructed in a clear conceptual framework on the basis of officially published IO tables in conjunction with national accounts and international trade statistics [21]. Since publication, it has proved very useful in analyses of global chain trade [22], domestic value-added content of gross exports [23], effects of trade policies [24], offshoring on labor demand [25] and numerous policy-oriented studies [26,27]. It is foreseeable that there will be more applications of WIOD in multiple research fields.

Furthermore, there are three kinds of data type in WIOD, which are World Input-Output Tables (WIOT), Regional Input-Output Tables (RIOT) and National Input-Output Tables (NIOT), and all of them are value-type IO data. In this paper, **NIOTs of China** were chosen, which provide IO tables of China in current prices expressed in millions of dollars and covers 35 industrial sectors from 1995 to 2011, to establish industrial complex networks and analyze development trends of Chinese macro industrial structure during recent two decades.

### Industrial Complex Network

**Fig 2:**
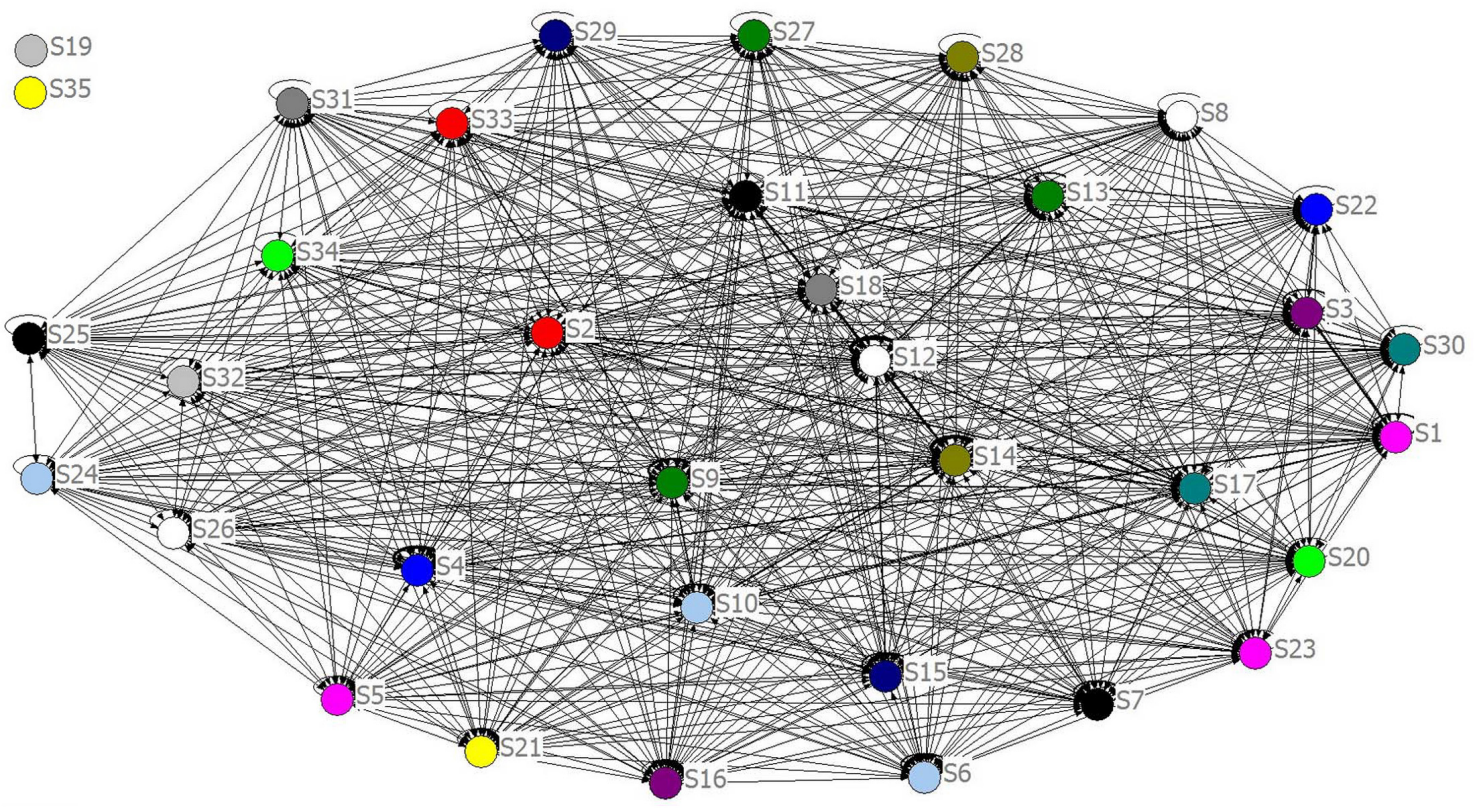
ISTN-CHN-2011 with Self-Loops.

In order to establish an industrial complex network, an industrial sector within a region was considered as a node, and the inter-industry IO relationship as a tie, whose weight represented the sale and purchase relationships between producers and consumers. Thus, we created a graph *G* = (*V*, *E*, *W*) containing *n* nodes, that here represent all industrial sectors within a nation or region, denoted by node set *V*. Pairs of nodes were linked by ties reflecting their interdependencies, constituting an asymmetric tie set *E*. However, in valued graphs, set *E* can actually be replaced by weight set *W*, which was extracted from the quadrant I of the basic matrix of the IO table in this paper. Taking into account that the properties of nodes must be consistent, the total output and input had been ignored temporarily, such as end-use and value-added, but intermediate inputs should be emphasized reflecting inter-industry relevance. Finally, this kind of model was named as **Industrial Shock Transmission Networks**, denoted by **ISTN**, since its purpose was to reflect how value stream and economic shock transfer in national or regional industrial system [28]. Adjacency matrices of 17 ISTN-CHN models are in S1 File. For instance, topological structure of ISTN-CHN-2011 is shown in Fig 2.

**Fig 3:**
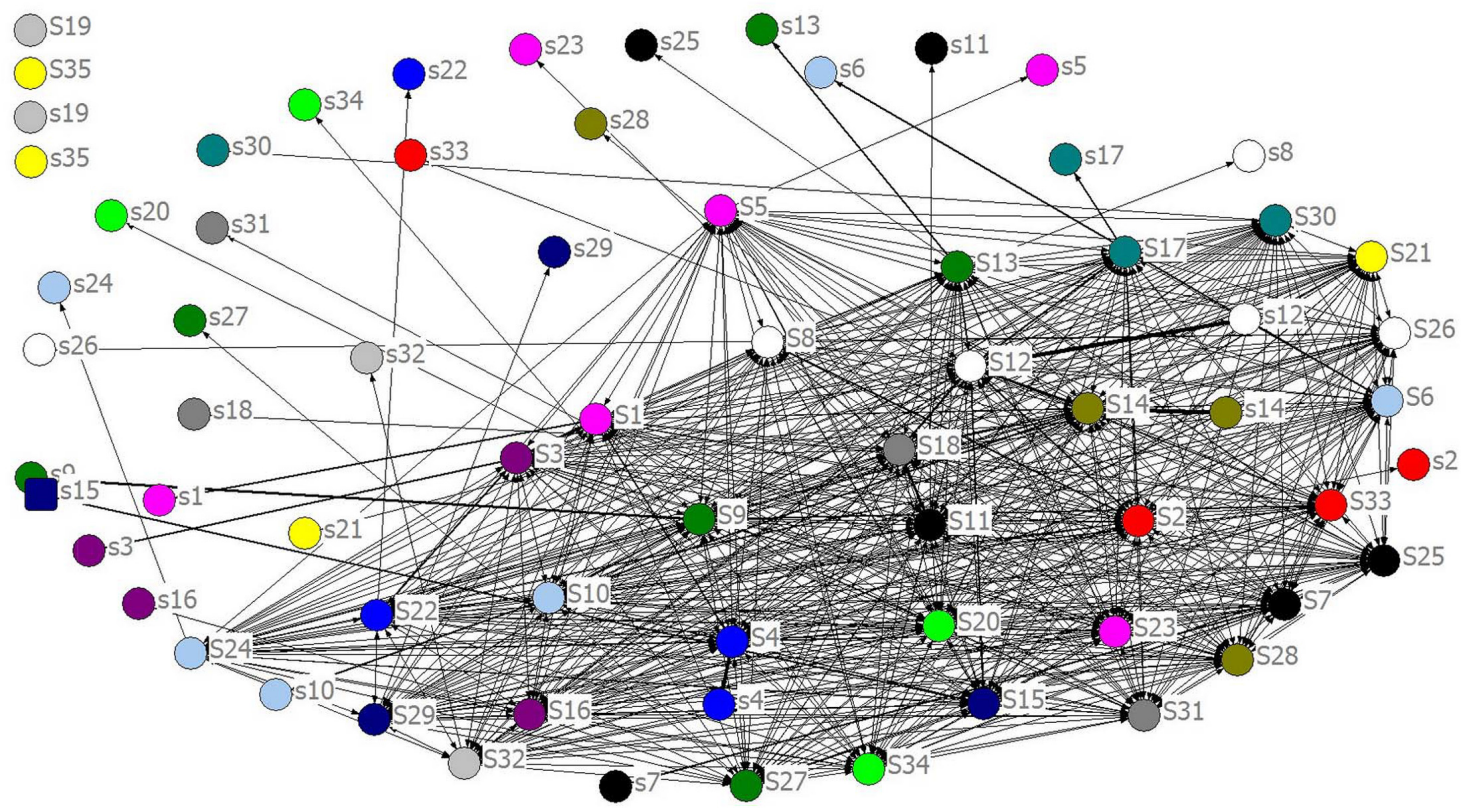
ISTN-CHN-2011 with Shadow Nodes.

From Fig 2 we can see that self-loops are allowed to exist in ISTN. In other words, *e*_*ii*_ or *w*_*ii*_ is likely to be non-zero. When exogenous variables change, value stream first affects its own before its diffusion according to the strength of the connection between the nodes. If many sectors consume a lot of its own products, there would be many self-loops with heavy weights in the network. For the sake of simplicity, self-loops are usually deleted in industrial complex networks. However, input of a sector on its own is close to or even more than half of its total output according to input-output table. For instance, China's *“Electrical and Optical Equipment”* sector’s proportion of investment on its own is more than 44.30% in 2011. So, self-loops are very important and non-ignorable. When calculating certain characteristics of network, for easy comprehension, self-loops were replaced by shadow nodes in this paper, denoted by small letter *s* as shown in Fig 3.

If some nodes’ in-degree (denoted by *k*^*in*^) or out-degree (denoted by $k_{i}^{out}$) are equal to zero, such as *S19*, *S35* in Fig 2 or *S19*, *S35*, *s19*, *s35* in Fig 3, it means they are isolate from the whole network and need to be removed when calculating characteristics.

In order to evaluate nodes’ contribution to transmit value stream, we applied betweenness and centrality in ISTN models. However, this kind of networks based on IO tables are different from general complex networks, because industrial sectors are almost completely connected, value stream spreads all over the industrial system, and self-consumption takes a great proportion of total out-put as mentioned above.

Thus, new betweenness and centrality measurement suitable for weighted and directed network should be introduced, then we could use them to study impacts of structural holes in context of network flow and random walk. Finally, Flow Betweenness proposed by Freeman and Random Walk Centrality proposed by Blöchl were chosen to evaluate industrial sectors’ long-term and short-term spreading effect respectively.

### Flow Betweenness

Freeman proposes **Flow Betweenness**, denoted by *C*_*F*_, to develop measures of centrality into weighted and directed network analysis. It is based on Fulkerson’s min-cut, max-flow theorem, which means the maximum flow from node *i* to *j* is exactly equal to the minimum cut capacity, that is, the smallest capacity of any of the *i* − *j* cut set [29]. Let *m*_*jk*_ be the maximum flow from a source node *i* to another sink node *k* and *m*_*jk*_(*i*) be the maximum flow from *j* to *k* that passes through node *i*. Then the degree to which the maximum flow between all unordered pairs of nodes depends on *i*, where *j* < *k* and *i* ≠ *j* ≠ *k*. The formula is:

$$C_{F}(i)=\sum_{j<k}\sum m_{jk}\left(i\right)$$ (1)

As the basis of *C*_*F*_, Ford and Fulkerson defined network flow is constrained by two additional conditions, one is the flow out of source node must be equal to the flow into sink node, another is the flow out of each intermediate node on any direct path linking adjacent nodes must be equal to the flow into relative sink one [30]. But, given these condition, ISTN models meet neither of them. The reason is, ISTN models don’t belong to closed network model because many raw materials, semi-finish products, finish products or various service not only transact in one national domestic economic system, but also parts of them export to other countries. In other words, gross output used by domestic industries is less than total use in consideration of regional integration of the world economy. Besides, industries consume parts of output made out by themselves, so the total input is neither equal to the total output of themselves.

Thus, when calculating normalized *C*_*F*_ by Ucinet 6.560, the result was beyond the limit of Ford and Fulkerson’s assumption. Moreover, the function of self-loops was transferred onto shadow nodes in order to cover more flow in network as possible, although *C*_*F*_ of new nodes located in the tail end of all paths was certainly zero.

### Random Walk Centrality

As one of basic dynamic process, the random walk is closely related with network research in many ways, especially, the nature of the network structure. Three indices are generally involved in studying random walk on networks. The first one is **First Passage Time**, denoted by **FPT**. After a source node released a walk signal, it will move to other adjacent nodes with equal probability or following certain transmission probability, thus the expected time to reach a pre-set sink node for the first time is FPT until repeating this process to arrive at it. According to FPT, the other two indices can be calculated. One is **Mean First Passage Time**, denoted by **MFPT**, which is equal to the arithmetic mean of all nodes in the network. Another is **Mean Absorption Time**, denoted by **MAT**, which is obtained by the average of FPT from each other nodes to a fixed sink node [31].

In view of progress of social network in recent years, Freeman's close centrality has been widely applied, generally denoted by *C*_*C*_. However, it restricts analyses on dense networks and nearly takes self-loops into account. Therefore, Blöchl introduced **Random Walk Centrality**, denoted by *C*_*RC*_, to describe how products and services flow within economic system.

As we know, the basic form of IO table is material-type, but the widely used is value-type. WIOT taken as modelling data belongs to the latter type, so inter-industry relationships are depicted by the flow of value stream. Borgatti proposes that money exchange process can be modelled as a Markov process, and the limiting probabilities for the nodes are proportional to degree [32]. But, in ISTN models, the similar process is more complex and need to be fully described by weights on edges. In other words, the transmission probability matrix of random walk, denoted by *M*(*i*, *j*), is subject to the impact of the importance of node *j*. In this paper, we defined *M* as follows:

$$M=S_{diag}^{-1}W$$ (2)

Where *W* is weight set or so-called weighted adjacency matrix, and *S*_*diag*_ is diagonal matrix consisting of nodes’ out-strengths $s_{i}^{out}$, that is $S_{diag}\left(i,i\right)=s_{i}^{out}$. For unweighted networks, *S*_*diag*_ can be instead of diagonal matrix *K*_*diag*_ directly [33]. Transmission probability matrix *M* describes possibilities when value stream transmits among product sectors by selecting next adjacent node as a path to continue, in the process of **Absorption Random Walk**, referred **ARW**. Hence *E*(*s*, *t*) is used to stand for MFPT, which is the expected number of steps when a random walk starts at source node *s* needs to reach sink node *t* for the first time. The formula is:

$$E\left(s,t\right)=\sum_{r=1}^{\infty }r\Pi_{s\to t}\left(r\right)$$ (3)

Where $\prod_{s\to t}\left(r\right)$ is the probability taking *r* steps from *s* to *t*. When *s* = *t*, $\prod_{s\to t}\left(r\right)=0$ and *E*(*t*, *t*) = 0. Even in undirected networks, *E*(*s*, *t*) is also asymmetric. This property reflects the fact that it is much more probable to pass by central intermediate nodes of a network than other ways.

According to the Random Walk Theory, when the random walk will no longer leave *t* after arriving at it, so before next transfer, it is supposed to modify the transmission matrix M by deleting its *t* row and column in order to form *M*_*−t*_.

In unweighted networks, paths between any different nodes are more likely to pass by central intermediate nodes with higher *C*_*C*_ values than surrounding ones with lower *C*_*C*_ values. Similarly, a weighted network such as ISTN based on the IO relationship, faster economic supply shocks tend to reach sensitive product sectors with higher *C*_*RC*_ values. Therefore, Blöchl defines random walk centrality as the inverse of the average MFPT by referring to Freeman’s closeness centrality. The formula is:

$$C_{RC}\left(i\right)=\frac{N}{\sum_{j=1}^{N}E\left(i,j\right)}$$ (4)

From Eq (4), it is clear that shorter MFPT taken to reach *i*, then higher its *C*_*RC*_ value will be. In addition, from this index’s derivation and calculation, it incorporates self-loops because they slow down the traffic between other nodes [34].

## Results

### Rank of Flow Betweenness

**Fig 4:**
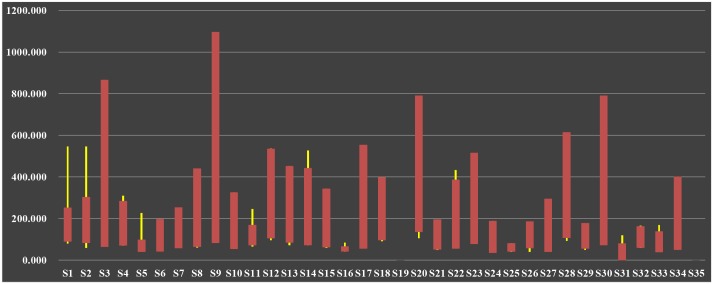
Candlestick of Flow Betweenness in ISTN-CHN from 1995 to 2011.

According to assumption and formula about *C*_*F*_, each industrial sector’s flow betweenness in ISTN-CHN models was calculated, and the results are in S2 File in details. For simplicity, its time-varying candlestick is shown in Fig 4.

When making candlestick, four columns of results were calculated, with initial value in 1995 and final value in 2011, maximum and minimum value during 1995 to 2011. Red cylinders represent initial value is smaller than final value, in which under-cutting is initial value and upper-cutting is final value. On the contrary, green cylinders represent initial value is bigger than final value, in which under-cutting is final value and upper-cutting is initial value. Besides, the upper end of yellow fine lines is maximum value during 1995 to 2011, and low end means minimum.

From Fig 4, it is obviously that *C*_*F*_ of each Chinese industrial sector has risen from 1995 to 2011, which means value stream transmitted by these sectors keeps continuous growth with annual increase of Chinese GDP. But, sectors’ growth degrees and variation trends are differential, for instance, some of them rise significantly or slightly, some rise first then decline, or alternatively. After all, evolution mechanism of industrial structure needs to be discovered out of these changes. Then, we carried out analyses about *C*_*F*_ in perspective of triple divisions of industrial sectors.

Results of *C*_*F*_ from 1995 to 2011 have shown that long-term spreading effect of *“Agriculture*, *Hunting*, *Forestry and Fishing”* (represented by node *S1*) in primary industries obviously subjects to time-varying fluctuation law. But, as downstream industry, *C*_*F*_ of *“Food*, *Beverages and Tobacco”* (represented by node *S3*) keeps grow all the time, and one of reasons may be that Chinese import ratio of agricultural products increases year by year, which makes it the biggest importing country for agricultural products in the world. Thus, no matter how domestic upstream supply side fluctuates, inputs from abroad keep downstream demand side steady increase. However, although ensuring domestic grain supply safety is crucial, the Chinese authorities must be aware of the potential industrial risk and implement industrial policies on promoting agricultural modernization, adjusting product price and protecting cultivated land resource. Besides, this phenomen occurs to *“Mining and Quarrying”* (*S2*), which means the rapid growth of Chinese manufacturing industries relies on imported minerals.

In ISTN-CHN models, nodes from *S4* to *S18* represent industrial sectors in secondary industries, in which distinct forward and direct relationships exist among certain adjacent sectors, in other words they respectively locate on upstream and downstream segments in industrial value chain. For instance, “*Coke*, *Refined Petroleum and Nuclear Fuel”* (*S8*) provides chemicals and raw materials to “*Chemicals and Chemical Products”*, “*Rubber and Plastics”*, and *“Other Non-Metallic Mineral”* (*S9*, *S10* and *S11*), and “*Basic Metals and Fabricated Metal”* (*S12*) provides various modern manufacturing industries with intermediate products. However, some phenomena are found in their variation trends of *C*_*F*_. On the one hand, *C*_*F*_ growth range of node *S8* is very large, in the meanwhile, nodes *S9* and *S10* supposed to be pulled up are lower than *S8*, for which reasons may be downstream sectors do not get motive power from upstream sectors effectively and upstream sectors’ production technique and process needs improving. On the other hand, *C*_*F*_ growth range of node *S11* is far from nodes *S13*, *S14* and *S15*, representing “*Machinery*, *Nec”*, “*Electrical and Optical Equipment”* and “*Transport Equipment”*, for which reasons may be downstream sectors’ urgent demand to intermediate products can’t be satisfied by domestic supply, so they need a great deal of import from other countries. Besides, *C*_*F*_ growth range of node *S12* matches to its downstream sectors, but Chinese steelworks cannot produce many standards of special steel and its products. In sum, Chinese industrial structure is still in process of heavy industrialization, and capital-intensive industries, such as coal, steel and oil, are on key developmental stage, so China as a large-scale manufacturing country needs science and technology to transform into powerful manufacturing country.

Tertiary industries’ connotation is much more complex, so in this paper we adopted Singlemann’s classification to distinguish their types, which include circulation services, producer servives, social services and personal services [35]. In circulation services, *C*_*F*_ growth range of “*Wholesale Trade and Commission Trade*, *Except of Motor Vehicles and Motorcycles”* and “*Inland Transport”* (*S20* and *S23*) are very large, which means they make great contribution to the development of economic society. In addition, “*Financial Intermediation and Renting of M&Eq”* and “*Other Business Activities”* (*S28* and *S30*) have the highest correlation with economic development level, and continuous increase of their *C*_*F*_ indicates they transmit more and more value stream for other sectors and facilitate Chinese industrialization.

### Rank of Random Walk Centrality

**Fig 5:**
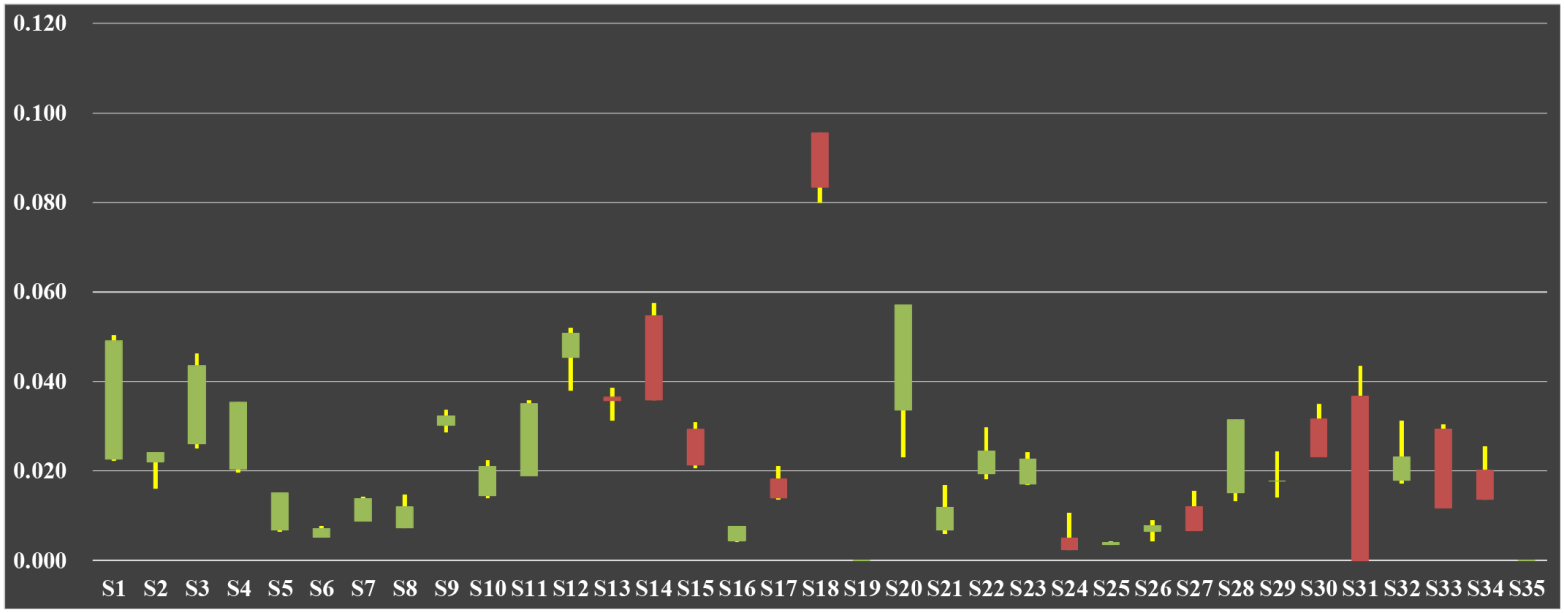
Candlestick of Random Walk Centrality in ISTN-CHN from 1995 to 2011.

According to assumption and formula on *C*_*RC*_, each industrial sector’s random walk centrality in ISTN-CHN models was calculated, and the results are in S2 File in details too. Its time-varying candlestick is shown in Fig 5.

*C*_*RC*_ measures short-term industrial spreading effect, and it depends on industrial structure. Thus, its quantitative value can be compared among different years, which means variation trends of industrial structure can be discovered by timing analysis. From Fig 5, *C*_*RC*_ of some Chinese industrial sectors rises and some declines from 1995 to 2011, and most of them are associated with varying degrees of fluctuation. Then, sectoral analyses are also carried out.

In ISTN-CHN models, *C*_*RC*_ of *“Agriculture*, *Hunting*, *Forestry and Fishing”* in primary sectors and its downstream *“Food*, *Beverages and Tobacco”* declines very distinctly after 1997. When we adopted NIOT to establish ISTN-CHN models, we pay more attention on domestic industrial structure of China without consideration of imported products. As the rapid growth of import ratio of agricultural goods, Chinese own agriculture has lost its crucial place in industrial value chain. In the meanwhile, capital flows to sectors with higher rate of return on investment. Thus, primary sectors’ transmission function has been weakened due to both import price shock and financial capital transfer.

*C*_*RC*_ of four sectors in secondary industries has large growth, including “*Electrical and Optical Equipment”*, “*Transport Equipment”*, “*Electricity*, *Gas and Water Supply”* and “*Construction”* (*S14*, *S15*, *S17* and *S18*). The former two belong to typical modern manufacturing industries and their status enhancement in industrial system benefits from higher participation in vertical specialization. The latter two are closely bound up with Chinese urbanization, especially construction, its production value share of GDP is getting higher with deepening impact on industrial structure, for which Chinese authorities should be alert for all kinds of consequent economic and social issues, such as subprime lending crisis and over-capacity.

As production and life pattern changing, traditional services sectors’ impact gradually decline for secular trend. According to variation trends of *C*_*RC*_, most of circulation services sectors’ short-term function of transmitting value stream go down, which indicates Chinese tertiary industries hit a bottleneck in process of transformation from labor intensive ones to capital intensive and technology intensive ones.

### Robustness Analysis

In ISTN models, industrial sectors locate at many value chains simultaneously, thus some of them play role of redundancy in the transmission process of value stream, which contribute a lot to the robustness of network structure. In order to prove sectors with higher *C*_*F*_ or *C*_*RC*_ are more important to economic system, we designed two sets of comparative experiment.

Firstly, we defined the connectedness of ISTN models, which represents the measurement of flow efficiency of value stream. In general network studies, **Distance** between *v*_*i*_ and *v*_*j*_ is equal to the minimum number of edges connecting them, and we call a path whose length is equal to distance the **Shortest Path**, denoted by *d*_*ij*_. If there is no such path between *v*_*i*_ and *v*_*j*_, it means *d*_*ij*_ = ∞. As to unweighted networks, we can calculate the **Average Path Length** (denoted by **APL**) of the whole network by Floyd algorithm, which depicts the degree of separation of nodes. But, as to weighted networks, especially when the similarity of nodes is proportional to their edge weight, it is infeasible to describe the feature of value stream flowing in economic system via APL. Thus, we must revise the classic Floyd algorithm, and make the new one capable of searching the path with the rapidest transfer and the lowest distortion of information, and we named it the **Strongest Relevance Path Length**, denoted by **SRPL**. The new iterative algorithm is:

$${\tilde{d}}_{ij}^{\left(k\right)}=\underset{i,j,k\in \left\{1,2,\ldots ,N\right\}}{\text{max}}\left\{{\tilde{d}}_{ij}^{\left(k-1\right)},\frac{{\tilde{d}}_{ik}^{\left(k-1\right)}{\tilde{d}}_{kj}^{\left(k-1\right)}}{{\tilde{d}}_{ik}^{\left(k-1\right)}+{\tilde{d}}_{kj}^{\left(k-1\right)}}\right\}$$ (5)

Where, ${\tilde{d}}_{ij}^{\left(k\right)}$ is SRPL between *v*_*i*_ and *v*_*j*_, actually representing an industrial value chain on which each edge is the tightest and path is the shortest. According to Eq (5), we can get SRPLs between any two of nodes, and they form a new matrix ${\tilde{D}}^{\left(N\right)}$:

$${\tilde{D}}^{\left(N\right)}=\left[\begin{array}{ccc} {\tilde{d}}_{11}^{\left(N\right)} & \cdots & {\tilde{d}}_{1N}^{\left(N\right)}\\ {\tilde{d}}_{21}^{\left(N\right)} & \cdots & {\tilde{d}}_{2N}^{\left(N\right)}\\ \vdots & & \vdots \\ {\tilde{d}}_{N1}^{\left(N\right)} & \cdots & {\tilde{d}}_{NN}^{\left(N\right)} \end{array}\right]$$ (6)

On the basis of ${\tilde{D}}^{\left(N\right)}$, we chose the average of its elements to measure the flow efficiency of value stream in ISTN models, and named it **Average Strongest Relevance Degree**, denoted by **ASRD**.

**Fig 6:**
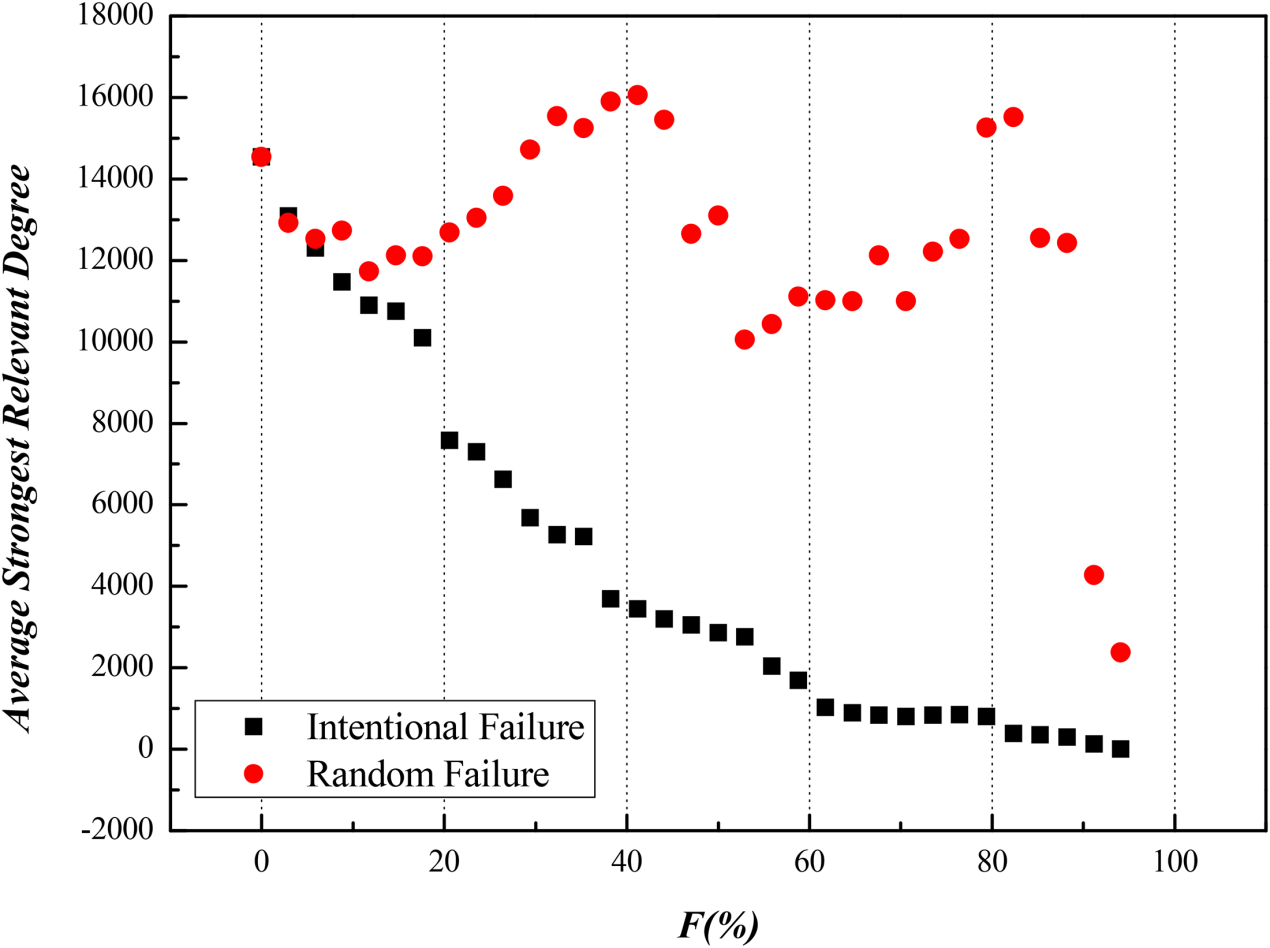
Impacts on ASRD Caused by Intentional Failure according to the Rank of Flow Betweenness in ISTN-CHN-2011.

**Fig 7:**
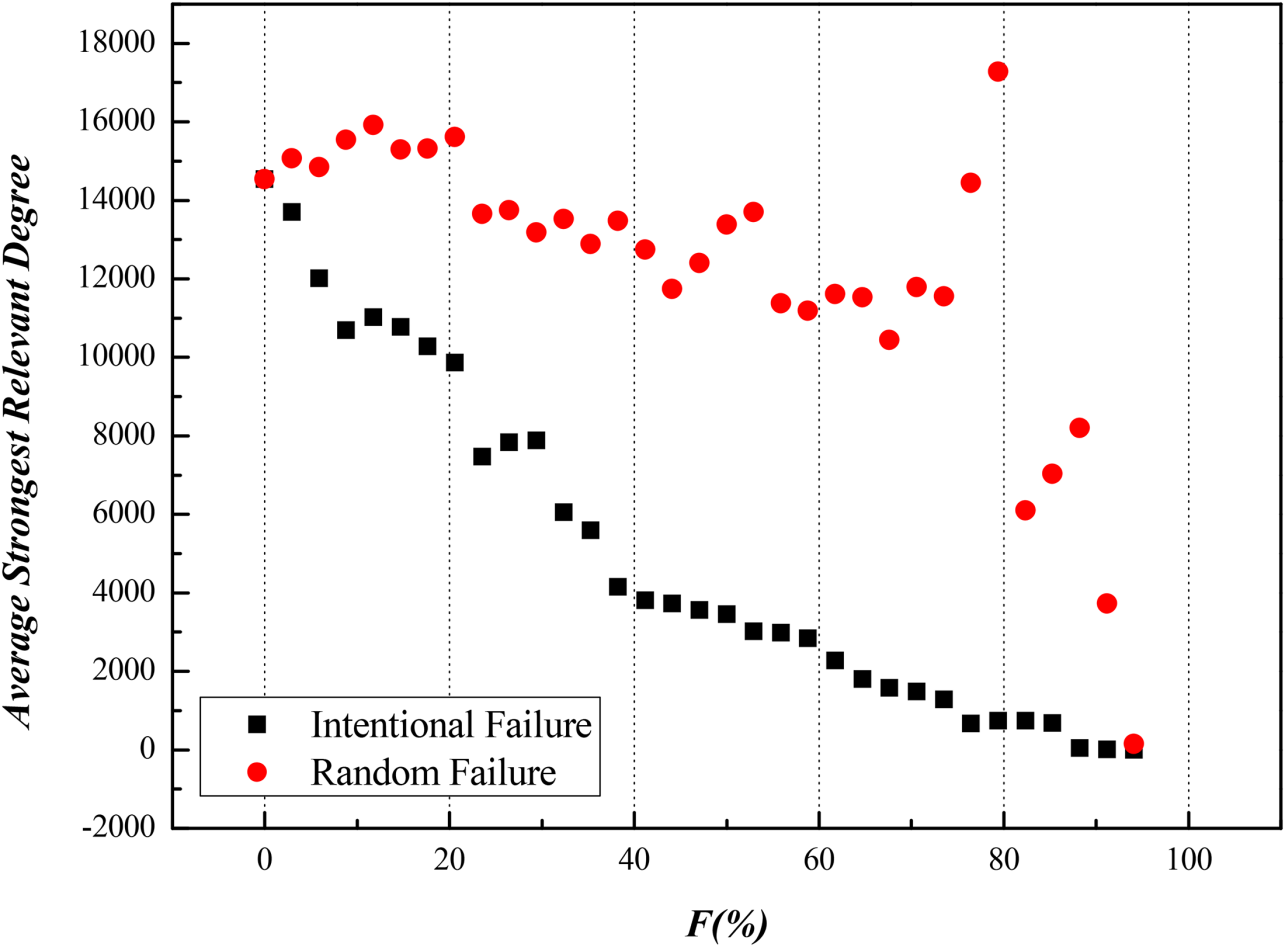
Impacts on ASRD Caused by Intentional Attack according to the Rank of Random Walk Centrality in ISTN-CHN-2011.

Secondly, we took ISTN-CHN-2011 for example and removed a part of nodes to observe what happens to ASRD of this model. In details, we intentionally removed specific nodes from those with higher *C*_*F*_ or *C*_*RC*_ to lower ones in one set of experiment, then we made random removal of nodes in another set of experiment as reference. After implementing intentional and random failure for both *C*_*F*_ or *C*_*RC*_, results are shown in Figs 6 and 7, which are in S3 File in details.

Finally, we can clearly see flow efficiency of value stream reduces by 50% when the ratio of nodes intentionally removed just reaches 20%, in the meanwhile, reference sets are far from this level of damage. Thus, on matter nodes with higher *C*_*F*_ or *C*_*RC*_, they play a leading role in transmission of value stream.

### Regression Analysis

**Fig 8:**
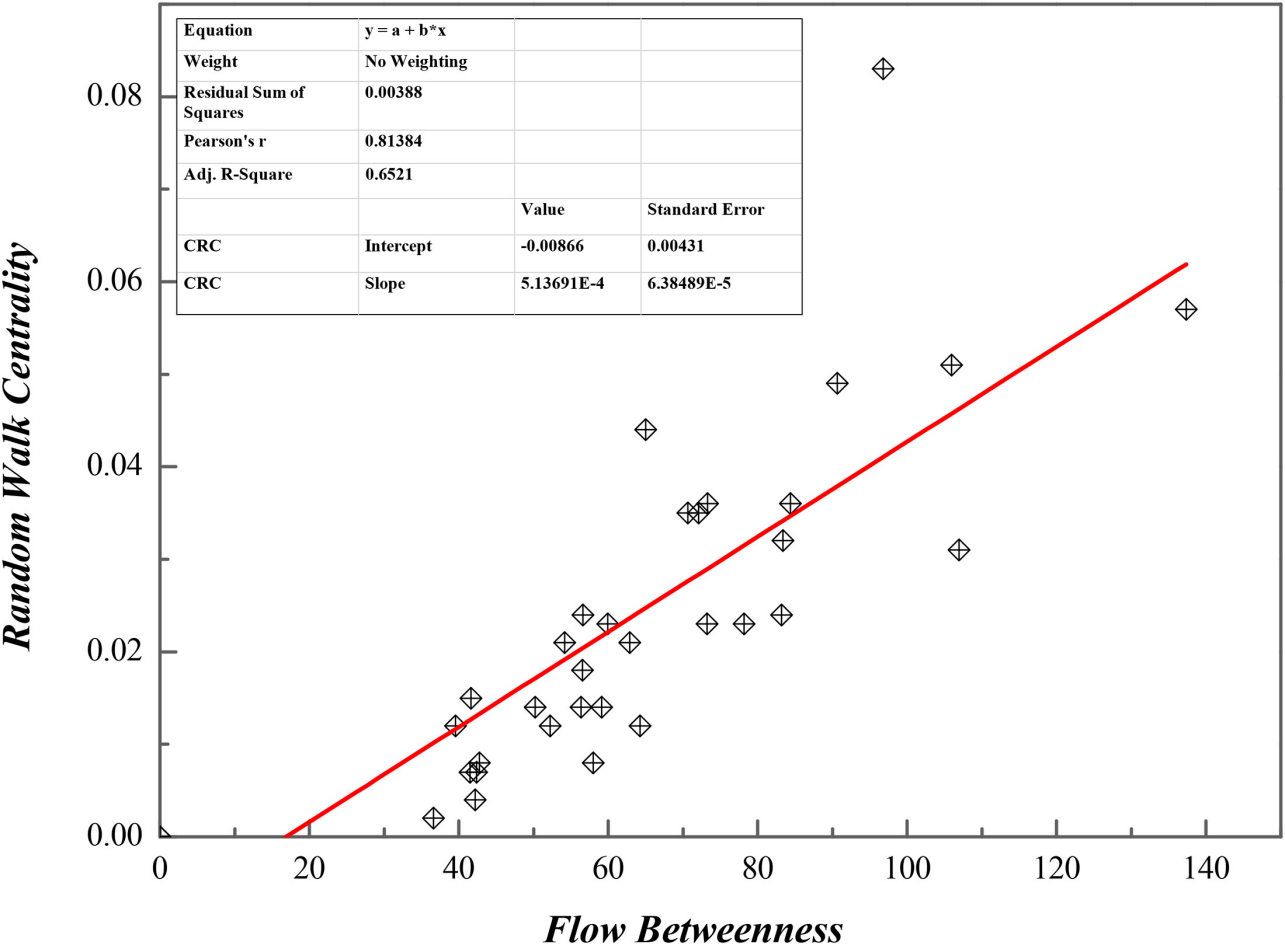
Regression Analysis on Flow Betweenness and Random Walk Centrality in ISTN-CHN-2011.

In order to carry out comparative analysis, a simple linear regression between *C*_*F*_ or *C*_*RC*_ was done, as shown in Fig 8, and data in details are in S4 File.

The results shows that and *C*_*F*_ or *C*_*RC*_ have a moderate linear correlation, which means that both of them have the same trend of increasing and decreasing, Adjusted R-squared is 0.65, so the relationship is reasonable.

According to fitting results, *C*_*F*_ or *C*_*RC*_ represent different spreading effect because of time-lag, which means there is a time process when certain industrial sector’s changes lead to other ones’. In other words, temporal span makes impacts on value stream transmission, in details, *C*_*F*_ can be treated as accumulative effect and *C*_*RC*_ as instantaneous effect as we already described in the above. However, *C*_*F*_ or *C*_*RC*_ are both restricted by relatively fixed industrial structure, so they are somewhat relevant to some extent. Thus, regression analysis needed to be implemented, and data required are in S5 File.

**Total Intermediate Consumption** (denoted by *TIC*) **and Total Output** (denoted by *TO*) are applied, in order to conduct multiple regression with *C*_*F*_. *TIC* is an accounting flow which consists of the total monetary value of goods and services consumed or used up as inputs in production by enterprises, including raw materials, services and various other operating expenses. *TO* is an economic concept used to measure total economic activity in the production of new goods and services in an accounting period. Therefore, these two indices respectively describe one sector’s capital in-flow and out-flow within economic system, and the regression results will indicate which flow determines long-term industrial spreading effect.

Firstly, Hausman test was adopted, and the results show that the cross-section random effect should be rejected under the 1% confidence level. After panel data fixed effect model, Durbin-Watson stat is 1.82, (1.58<D.W. = 1.82<2.42), which shows the null hypothesis should be accepted, the disturbance term does not exist a first-order positive autocorrelation, and the higher R-squared means the goodness-of-fit of model is stronger. Then, stepwise regression was carried out, finding that *TO* has negative affect, so the final panel data fixed effect model can be expressed as follows:

$$\begin{array}{l} {\text{C}}_{Ft}=\;56.25708+0.260025{\text{C}}_{Ft-1}+0.000312\text{TIC}\\ \;\;\;\;\;\;\;\;\;\;\;\;\left(0.0000\right)\;\;\;\;\;\;\left(0.0000\right)\;\;\;\;\;\;\;\;\;\;\;\;\;\left(0.0000\right) \end{array}$$ (7)

All of the coefficients are significant at 1% confidence level. Eq (7) displays that *C*_*F*_ has positive correlation with its first-order lag, which means every 1 unit of last year will make 0.260025 impact on this year, and *TIC* also has positive affect, which contribute 0.00312 to *C*_*Ft*_ over the same period.

Similarly, **Induction Coefficient** (denoted by *ISD*), and **Influence Coefficient** (denoted by *IPD*) were also introduced for multiple regression with *C*_*RC*_. *ISD* means in the national economy, when all industries are adding a unit of final use, thereby subjects to the needs of an industry level sensors. *IPD* refers to a national increase of one unit of end-use industries, the right of the national economy resulting from the production needs of industry, affects the degree of correlation coefficient. According to their definition, they respectively evaluate one sector’s sensitivity and extraversion to industrial structure, and the regression results will show which index determines the function of short-term industrial spreading effect.

After the same estimation process, the fixed effect panel data model was used again, and *IPD* has negative affect, so the final panel data fixed effect model can be expressed as follows:

$$\begin{array}{l} {\text{C}}_{RCt}=\;-0.0000519+0.862493{\text{C}}_{RCt-1}+0.003041\text{ISD}\\ \;\;\;\;\;\;\;\;\;\;\;\;\;\left(0.9589\right)\;\;\;\;\;\;\;\;\;\;\;\left(0.0000\right)\;\;\;\;\;\;\;\;\;\;\;\;\;\left(0.0044\right)\;\; \end{array}$$ (8)

Durbin-Watson stat is 1.76, according to D.W. Test table (1.58<D.W. = 1.76<2.42), which means that the null hypothesis should be accepted, the disturbance term does not exist a first-order positive autocorrelation. Also the higher R-squared means the goodness-of-fit of model is very strong. Eq (8) displays that *C*_*RC*_ has positive correlation with its first-order lag, which means every 1 unit of last year will have 0.862493 impact on this year.

In sum, exogenous and endogenous variables jointly influence industrial structure and make it change slowly over time, Thus, *C*_*F*_ and *C*_*RC*_ are relevant with their last annual status. In addition, industrial spreading effect is decided by capital in-flow and its sensitivity, not out-flow or extraversion. So, both long-term and short-term value stream transmission depend on how many products and services sectors can get. If they could get enough stuff in economic system, represented by huge intermediate consumption, they are to be regarded as brokers with bigger information superiority and more intermediate interests according to structural holes theory, which means their position and function are more important in the economic system than the others.

## Conclusions

Industry analyses are concentrating more on dynamic swarm than static individual. IO analysis, as an important research tool, focuses more on static analysis. However, the fundamental aim of industry analyses are to figure out how interaction between different industries makes impacts on economic development, which turns out to be a dynamic process. So, in this paper, ISTN models were established based on IO tables as one bridge connecting accurate static quantitative analysis and comparable dynamic one.

Based on revised structural holes theory, it is obvious that industries with bigger flow betweenness or random walk centrality would bring about more intensive industrial spreading effect to the industrial chains they stands in. If they suffered from intentional failure, the connectedness of ISTN models will decline rapidly. And according to results of multiple regression, value stream transmission of industrial sectors depends on how many products and services sectors they can get from the others, and these sectors are regarded as brokers with bigger information superiority and more intermediate interests.

But, results from this paper are not enough for forecasting evolutionary trends of industrial structure. In that, past years’ IO data were adopted to depict variation trends of two kinds of betweenness and centrality as well as explain dynamic mechanics of industrial effect spreading over different temporal span. However, it is necessary to accurately estimate industrial development in consideration of multiple exogenous variables’ effect, and there will be big difference when different exogenous variables act on different industrial sectors. Thus, in following studies, important exogenous variables will be chosen for analogue simulation, evolutionary trends of industrial structure will be forecasted according to industrial environmental changes by scenario analysis, such as link prediction, and industrial policy to specific sectors are supposed to be proposed for promoting its scientific development.

## Supporting Information

S1 File Adjcacency Matrix of ISTN-CHN Models. (XLSX)

S2 File Rank of Flow Betweenness and Random Walk Centrality. (XLSX)

S3 File Robustness Analysis. (OPJ)

S4 File Regression Analysis. (OPJ)

S5 File TIC&TO&ISD&IPD in Regression Analysis. (XLSX)
